# REDEFINING SUCCESSFUL AGING: INTEGRATING DISABILITY AND THE ROLE OF MODIFIABLE FACTORS

**DOI:** 10.1093/geroni/igae098.3704

**Published:** 2024-12-31

**Authors:** Madina Khamzina, Shannon Mejia, Wendy Rogers

**Affiliations:** University of Maryland College Park, Silver Spring, Maryland, United States; University of Illinois Urbana-Champaign, Urbana, Illinois, United States; University of Illinois Urbana-Champaign, Champaign, Illinois, United States

## Abstract

Disability is a common experience in older adulthood, yet traditional models often neglect its role in successful aging. This study integrates disability into the successful aging framework, utilizing subjective well-being and self-rated health as key indicators. We aim to link disability and successful aging and examine modifiable factors that could foster successful aging among individuals with disabilities over time. We conducted cross-sectional analyses using data from two year periods, the 2011 (N=6,440) and 2020 (N=3,220) waves of the National Health and Aging Trends Study. Disabilities related to mobility, hearing, and vision were self-reported. Indicators of successful aging included subjective well-being and self-rated health. Physical activity and social engagement were examined as modifiable protective factors. Moderation analyses tested whether these factors had stronger effects among individuals with disabilities. The prevalence of successful aging among those with disabilities was 40% in 2011 and 38% in 2020. Regression analyses showed that each type of disability was negatively associated with successful aging indicators. Social engagement and physical activity consistently supported successful aging across both time periods. Social engagement was especially beneficial in 2011 for those with mobility, hearing, and multiple disabilities. Findings underscore the importance of recognizing successful aging with disability and highlight the role of modifiable factors like physical activity and social engagement in promoting successful aging. This study supports the inclusion of disability in aging paradigms, emphasizing adaptive strategies for older adults with disabilities.

